# Pupillary dysfunction during hypothermic circulatory arrest: insights from automated pupillometry

**DOI:** 10.1186/s13054-023-04490-x

**Published:** 2023-05-22

**Authors:** Lorenzo Peluso, Federica Baccanelli, Valentina Grazioli, Paolo Panisi, Fabio Silvio Taccone, Giovanni Albano

**Affiliations:** 1 Department of Biomedical Sciences, Humanitas University, Via Rita Levi Montalcini, 4, 20072 Pieve Emanuele, Milan, Italy; 2 Department of Anesthesia and Intensive Care, Humanitas Gavazzeni, Via M. Gavazzeni, 21, 24125 Bergamo, Italy; 3 Department of Cardiac Surgery, Humanitas Gavazzeni, Via M. Gavazzeni, 21, 24125 Bergamo, Italy; 4 Department of Intensive Care, Hopital Erasme – Université Libre de Bruxelles, Route de Lennik, 808, 1070 Brussels, Belgium

Dear Editor,

A low Neurological Pupil Index (NPi) value, as measured by automated pupillometry, has been linked to notable prognostic significance following anoxic brain injury [1]. Despite minimal interference from sedatives and opioids, instances of low NPi accompanied by intact neurological recovery have been reported in conjunction with severe hypercapnic acidemia or the administration of inhaled sevoflurane and ketamine [2]. In the setting of cardiac surgery, no data are available on the prognostic value of low NPi values; circulatory arrest, selective antegrade cerebral perfusion (SACP) and hypothermia may lead to significant changes in brain physiology and, therefore, of pupillary function. Whether abnormal NPi can indicate severe brain injury during cardiac surgery remains unknown.

We report the case of a 42-year-old male, who underwent a planned aortic arch replacement due to the progression of a previously treated type A ascending aortic dissection. The prior dissection had resulted in paresis of the left upper extremity. Anesthesia was conducted using propofol (6–10 mg/kg*h), fentanyl (total dose: 400 mcg) and rocuronium; in the operating room, neuro-monitoring included bilateral frontal processed EEG and regional oxygen saturation (rSO_2_; SEDLINE and O3, Masimo, Irvine, CA, USA) and automated pupillometry (Neuroptics NPi-200, Laguna Hills, CA, USA), as shown in Table 1. After the induction of anesthesia, left and right rSO_2_ values were 62 and 67%, respectively; patient state index (PSI) value was 40; and left and right NPi values were 4.8 and 4.7, respectively. Cardiopulmonary bypass was initiated following the insertion of an arterial cannula into the previously implanted ascending aorta prosthesis and the placement of a venous cannula into the right atrium. Moderate hypothermia was induced, reaching a core body temperature of 26.0 °C, and circulatory arrest was commenced prior to the aortic arch replacement.

**Table 1:** Summary of different neurophysiological parameters, according to different body temperatures

	Temperature (°C)	Propofol dose (mg/kg/h)	Left NPi	Right NPi	Left pupillary size (mm)	Right pupillary size (mm)	PSI	Left rSO_2_ (%)	Right rSO_2_ (%)
After induction	35.9	8	4.8	4.7	2.40	2.04	40	62	67
SACP	26.0	4	1.2	1.5	4.14	3.97	1	58	59
Rewarming	28.3	3	4.0	4.0	1.52	1.37	4	56	58
End of surgery	35.2	2	4.1	4.8	1.74	1.66	24	58	61
12-h	36.1	2	4.2	4.7	4.69	3.66	–	–	–
24-h	35.8	–	4.6	4.8	1.86	1.99	–	–	–

*SACP* Selective antegrade cerebral perfusion; *NPi* Neurologic Pupil Index; *PSI* Patient state index; *rSO*_*2*_ Regional brain saturation

After the placement of SACP, left and right rSO_2_ were 58 and 59%, respectively; the PSI value was 1 (i.e., suppressed EEG), and left and right NPi were 1.2 and 1.5, respectively. During the rewarming phase, left and right NPi increased to 4.0 and 4.0 at 28 °C, with an EEG still suppressed, and eventually to 4.1 and 4.8, respectively, at 35 °C. Following the surgery, the patient was admitted to the intensive care unit (ICU) and successfully weaned from mechanical ventilation after 16 h. No postoperative delirium or new neurological dysfunction was observed. On the third day of ICU admission, the patient was transferred to the cardiac surgery ward for continued care.

This represents the first case report of extremely low NPi values observed during hypothermic circulatory arrest for aortic arch surgery, without any associated brain injury. The relationship between hypothermia and the observed results remains uncertain. As NPi values are insensitive to propofol and fentanyl [2], the substantial decrease in NPi values might be attributed to either reduced body temperature or suppressed neuronal activity. However, the NPi increased while the EEG background remained suppressed, suggesting that hypothermia is the most plausible explanation for our findings. Hypothermia is known to cause an increase in pupillary size with bilateral mydriasis; however, these results were obtained in healthy individuals undergoing eye surface cooling and no NPi was assessed [3]. In cardiac arrest survivors treated with hypothermia at temperatures between 33 and 36 °C, low NPi values were associated with other predictors of poor outcomes, implying that abnormal NPi may be indicative of brain injury within this range of body temperatures [4]. During cardiac surgery without profound hypothermia and SACP, pupil dilatation (17–53%) was observed, which was unaffected using fentanyl [5].

Our report challenges the clinical significance of assessing pupillary function during cardiac surgery with hypothermic circulatory arrest. Further studies are needed to determine whether monitoring pupillary function is an accurate way to identify patients at risk of brain injury in this setting or if other monitoring tools, such as EEG or non-invasive oxygen saturation, may be more appropriate. Although we did not report specific data on manual pupillary assessment, it remains less accurate than automated pupillometry [1], which should be the preferred method whenever pupillary assessment is necessary. Prospective studies are needed to establish the strengths and limitations of automated pupillometry and NPi in this context, enabling more accurate neurological evaluation in hypothermic patients undergoing procedures such as aortic arch surgery.

## Data Availability

The datasets used and/or analyzed during the current study are available from the corresponding author on reasonable request.

## Abbreviations

NPi: Neurological Pupil Index
SACP: Selective antegrade cerebral perfusion
EEG: Electroencephalography
PSI: Patient state index
ICU: Intensive care unit
